# Machine Learning and Digital Biomarkers Can Detect Early Stages of Neurodegenerative Diseases

**DOI:** 10.3390/s24051572

**Published:** 2024-02-29

**Authors:** Artur Chudzik, Albert Śledzianowski, Andrzej W. Przybyszewski

**Affiliations:** 1 Polish-Japanese Academy of Information Technology, Faculty of Computer Science, 86 Koszykowa Street, 02-008 Warsaw, Poland; artur.chudzik@pjwstk.edu.pl (A.C.); albert.sledzianowski@pjwstk.edu.pl (A.Ś.); 2 UMass Chan Medical School, Department of Neurology, 65 Lake Avenue, Worcester, MA 01655, USA

**Keywords:** neurodegenerative diseases, Alzheimer’s disease, Parkinson’s disease, digital endpoints, online cognitive testing, eye-tracking, machine learning, early detection, digital phenotyping

## Abstract

Neurodegenerative diseases (NDs) such as Alzheimer’s Disease (AD) and Parkinson’s Disease (PD) are devastating conditions that can develop without noticeable symptoms, causing irreversible damage to neurons before any signs become clinically evident. NDs are a major cause of disability and mortality worldwide. Currently, there are no cures or treatments to halt their progression. Therefore, the development of early detection methods is urgently needed to delay neuronal loss as soon as possible. Despite advancements in Medtech, the early diagnosis of NDs remains a challenge at the intersection of medical, IT, and regulatory fields. Thus, this review explores “digital biomarkers” (tools designed for remote neurocognitive data collection and AI analysis) as a potential solution. The review summarizes that recent studies combining AI with digital biomarkers suggest the possibility of identifying pre-symptomatic indicators of NDs. For instance, research utilizing convolutional neural networks for eye tracking has achieved significant diagnostic accuracies. ROC-AUC scores reached up to 0.88, indicating high model performance in differentiating between PD patients and healthy controls. Similarly, advancements in facial expression analysis through tools have demonstrated significant potential in detecting emotional changes in ND patients, with some models reaching an accuracy of 0.89 and a precision of 0.85. This review follows a structured approach to article selection, starting with a comprehensive database search and culminating in a rigorous quality assessment and meaning for NDs of the different methods. The process is visualized in 10 tables with 54 parameters describing different approaches and their consequences for understanding various mechanisms in ND changes. However, these methods also face challenges related to data accuracy and privacy concerns. To address these issues, this review proposes strategies that emphasize the need for rigorous validation and rapid integration into clinical practice. Such integration could transform ND diagnostics, making early detection tools more cost-effective and globally accessible. In conclusion, this review underscores the urgent need to incorporate validated digital health tools into mainstream medical practice. This integration could indicate a new era in the early diagnosis of neurodegenerative diseases, potentially altering the trajectory of these conditions for millions worldwide. Thus, by highlighting specific and statistically significant findings, this review demonstrates the current progress in this field and the potential impact of these advancements on the global management of NDs.

## 1. Introduction

Aging is a significant risk factor for neurodegenerative diseases (NDs) such as Alzheimer’s (AD) and Parkinson’s (PD), despite advancements in technology that have improved our quality of life and longevity [1,2,3]. Unfortunately, the complexity of the disease process, involving various contributing factors, presents a challenge in identifying effective remedies [4].

The complexity of NDs lies in a spectrum of disorders characterized by a primary loss of cells, leading to secondary cell loss in other brain regions [5]. Processes correlated with AD begin over 30 years, whereas cognitive changes begin over about 15–11 years, before the first AD symptoms [6,7]. Unfortunately, the prevalence of Alzheimer’s Disease-related dementia is fast increasing due to our aging population [8].

Sadly, the prevalence worldwide is estimated to be as high as 24 million; by 2050, the AD number could rise to 139 million worldwide [9]. Currently (Q1′24), there is no cure for AD, as during the first clinical symptoms and neurological diagnosis many parts of the brain are already affected without the possibility to recover.

The second (after AD) most common neurodegenerative disease is Parkinson’s Disease (PD). This disease is characterized mainly by motor but also by cognitive disorders [10]. The prevalence of Parkinson’s Disease is expected to increase significantly by 2050, with estimates suggesting a doubling of the current number of affected individuals. This is due to a combination of factors, including an aging population, declining smoking rates, and increasing industrialization [11,12]. The economic burden of the disease is also projected to rise, with the cost of medical expenses and indirect costs such as reduced employment expected to increase substantially [11]. These projections highlight the urgent need for innovative treatments and a coordinated global response to address the growing impact of Parkinson’s Disease.

It is noteworthy that both Alzheimer’s Disease and Parkinson’s Disease are neurodegenerative diseases characterized by substantial and irreversible neuronal loss, however in different regions of the brain. Commonly, neurodegeneration begins two to three decades before observed symptoms. Hence, the best chance to fight NDs is to estimate the beginning period of the ND-related brain changes [13].

Recent research on the beginnings of NDs has focused on the role of molecular biomarkers, such as miRNAs (small gene regulators) and exosomes (tiny cellular messengers). It has shown promise in detecting neuronal dysfunction in the presymptomatic stage of NDs [14]. These biomarkers, along with other laboratory and biochemical markers, are being explored for their potential in early diagnosis and disease progression assessment [15]. However, the use of analog biomarkers in Alzheimer’s Disease diagnosis has limitations, including the need for biological samples and hospitalization [16]. 

Despite these challenges, the development of bioassays (sensitive biological detection tests) and the identification of biological markers in blood, plasma, and serum have shown promise in overcoming these limitations [17]. However, the validation of the clinical usefulness of these biomarkers is still incomplete, and further research is needed to standardize their readout and evaluate their performance in detecting early disease [18]. 

Therefore, the search for an ideal biomarker for Alzheimer’s and Parkinson’s Disease continues, with the goal of achieving a reliable and accurate diagnosis at the earliest clinical stages. Analog biomarkers, despite their strengths, hold limitations as well. Conversely, digital technologies, which provide objective, high-frequency data, are being considered as a solution to the current subjective measures of NDs [19]. 

These tools, including AI and remote sensing technologies, are promising avenues for early detection and monitoring of neurodegenerative diseases that can be deployed non-invasively and potentially at scale [20,21]. Yet, there is an ongoing discussion regarding the connection between digital and analog biomarkers and their correlation. This is a gap in the research that still calls for interdisciplinary validation, hence posing a challenge between the medical, IT, legal, and ethical fields.

For that reason, this review aims to provide an interdisciplinary perspective and highlight the challenges in the intersection between technology and medicine, based on recent findings. 

## 2. Biological Definition and Hidden Nature of NDs 

Digital biomarkers emerged from analog biomarkers [22]. Hence, to properly interpret the outcomes of digital tools, it is important to understand first the biological definitions of both diseases, their measurement parameters, their relevance to neurodegenerative diseases, and how AI can enhance the interpretation and application of these biomarkers.

Therefore, we define NDs as a group of diseases that cause the progressive loss of structure or function of neurons, including death of neurons. Despite the fact that Alzheimer’s and Parkinson’s are in the same cluster of disorders, they exhibit distinct biological characteristics [23]. 

For example, in Parkinson’s Disease, by the time symptoms like *bradykinesia* or tremor became apparent, between 30 and 70% of the substantia nigra (SN—an area of the midbrain) is already irreparably damaged, limiting the actionable time window. The range of 30–70% is commonly cited, but this can vary based on individual cases and the methods used for measurement. As a consequence of this damage, we lose SN neurons, the so-called dopaminergic neurons of the midbrain, which are the main source of dopamine (DA) in the central nervous system (CNS) [24]. 

Moreover, the impact of neurodegeneration can be noticeable in eye movements (EMs). This is because the substantia nigra influences eye movements among its many roles in movement regulation. The SN helps regulate the action of the superior colliculus (SC) by providing inhibitory input. The SC initiates reflex orienting by sending control signals to the gaze centers in the midbrain, and this process is impaired by the loss of neurons in the SN.

In the context of Alzheimer’s Disease, the main affected part of the brain is a region responsible for memory and spatial navigation. AD onset is primarily linked to changes in the hippocampus, which rapidly loses tissue and functional connectivity with other brain regions, leading to atrophy and cognitive impairment [25]. Changes in the hippocampus, including altered neurogenesis, are early events in AD and may worsen memory impairment [26]. Spatial disorientation, a common symptom of AD, is linked to changes in the medial temporal and parietal brain regions [27]. These changes in the hippocampus and associated brain regions are associated with altered gene expression, synaptic excitability, and plasticity, which contribute to memory loss in AD [28].

However, thanks to internal compensation mechanisms, the neurodegeneration that occurs in the meantime is hidden from the outside. This hidden progression of the disease makes early detection a challenge in research, which can hinder the development of prevention strategies [29,30]. 

## 3. Biological Definition Determines Parameters for Digital Measurements

There are three kinds of symptoms in NDs, related to different structures in the brain being affected by the disease: cognitive (primarily in AD, secondarily in PD), motor (primarily in PD, less evident in AD), and emotional (observed in both, but characteristic for late-onset AD). Sadly, research has identified a range of non-motor symptoms (NMSs) as well, including cognitive problems, apathy, depression, anxiety, hallucinations, and psychosis, as well as sleep disorders, fatigue, autonomic dysfunction, sensory problems, and pain [31]. Importantly, symptoms can occur in the pre-motor phase of the disease and are not fully addressed by current treatments [32].

To better sense the hidden and ongoing changes in the brain, the intersection of technology and medicine stands out as a perspective direction, especially with the opportunity for wide application that can help in prevention strategies. The importance of those detectors, sensitive enough to catch invisible signs of the disease, is repeatedly underlined in the literature [20,33]. 

Fortunately, as we present, researchers analyzing NDs have become interested in changes in emotional, cognitive, and EM patterns, deeply examining their parameters for building AI models for disease classification.

Given the narrative nature of our review, our aim is to provide a broad overview rather than a detailed technical analysis. However, recognizing the importance of precision in terminology, we have reviewed the manuscript to ensure that each mention of AI and ML is contextually accurate, clarifying the distinction between the broader field of AI and the specific application of ML as a subset of AI techniques.

## 4. Methods

This narrative review synthesizes existing research on the integration of machine learning and digital biomarkers in the diagnosis and monitoring of neurodegenerative diseases. We explore the advancements, challenges, and future directions in this interdisciplinary field, emphasizing the potential of these technologies to support early detection and patient care. The scope encompasses studies related to eye tracking, facial expression analysis, cognitive testing, and other digital phenotyping tools in the context of diseases such as Alzheimer’s and Parkinson’s.

To capture a comprehensive body of literature, we conducted searches in major scientific databases, including Google Scholar, IEEE Xplore, and PubMed. Our search was supplemented by the use of research tools like Litmaps and Mendeley, which helped in mapping the literature connections and identifying key contributions.

We utilized a combination of keywords and phrases such as “eye tracking”, “facial expressions”, “cognitive testing”, “machine learning,” “digital biomarkers,” “digital phenotyping,” “neurodegenerative diseases,” “Alzheimer’s Disease,” and “Parkinson’s Disease.” Boolean operators (AND, OR) were used to refine the search, ensuring a focused retrieval of relevant studies.

Our selection process included a diverse range of studies that provide insight into the development and application of digital biomarkers and machine learning in the context of neurodegenerative diseases. We cite articles published between 1937 and 2023, written in English, and focusing on original research. The selection was guided by the relevance of each article to the review’s themes, the innovative use of technology in neurodegenerative disease diagnostics, and the potential impact on clinical practices and patient outcomes.

The literature was synthesized to highlight key findings, identify thematic trends, and discuss the implications of integrating digital biomarkers with machine learning for neurodegenerative disease diagnostics. We analyzed the selected studies in the context of their contribution to advancing technology, addressing challenges such as data accuracy, privacy concerns, and the need for validation, as well as their potential for clinical application. This thematic analysis allowed us to draw insights into the current state of the field, identify gaps in the literature, and propose directions for future research.

The review integrates findings from the selected literature into a cohesive narrative assessment, discussing the evolution of digital biomarkers and machine learning in the medical field, with a particular focus on neurodegenerative diseases. By examining the studies through a thematic lens, it provides a comprehensive overview of the landscape, including the technological advancements, methodological challenges, and the ethical considerations of implementing these technologies in clinical settings.

## 5. Remote Oculomotor and Facial Expression Analysis Indicate Progression of NDs

Eye tracking, an established technique for measuring eye movements, plays a crucial role in understanding neurodegenerative diseases. This technique is employed to record the paths of eye movements, often under controlled conditions such as the “follow the green dot on the screen” task. Camera-based eye trackers are the most common form, but other methods like electrooculography (EOG) are available as well [34]. Eye movement disorders offer a window into early changes in brain computation, especially as they are affected early in neurodegenerative diseases [35]. By studying these movements, researchers can gain insights into how these diseases impact the brain’s functionality. 

Additionally, when combined with facial expression analysis, eye tracking becomes even more powerful [36]. These methodologies are being studied in various contexts of neurodegenerative diseases to identify early signs and progression markers. The combination of eye tracking and facial expression analysis offers a comprehensive approach to studying NDs, providing a more nuanced understanding of the diseases. 

## 6. Eye Tracking Helps to Determine the Disease Probability 

Eye tracking technology has been utilized to differentiate between Parkinson’s Disease patients and healthy controls, with promising results. For example, in a recent (2023) study, Brien et al. collected video-based eye tracking data on an interleaved pro/anti-saccade task of 104 PD patients and 106 healthy controls [37]. They used features of saccades, pupil, and blink behavior to predict confidence scores for PD/PD-MCI/PDD diagnosis with the Linear Mixed Model to determine the disease probability by different eye tracking biomarkers. This classifier reached a sensitivity of 0.83 and a specificity of 0.78 and the Receiver Operator Characteristic Area Under the Curve (ROC-AUC) of the classifier was 0.88. The predicted confidence scores were indicative of PD motor and cognitive performance scores. The study’s findings demonstrate that eye tracking biomarkers can reliably indicate PD motor and cognitive performance scores, offering a non-invasive method for early disease detection.

Similarly, other studies have explored eye tracking for disease detection. Bejani et al. (2022) also utilized video-based eye tracking to analyze smooth pursuit eye movements in PD patients and controls [38]. A collection of parameters was obtained, including complexity measures based on entropy/regularity, describing the system’s dynamic and features for assessing self-similarity. The Support Vector Machine (SVM) was used for classification and for the PD and control groups an accuracy of 0.74 was obtained (sensitivity 0.73, specificity 0.74). This research further supports the idea that eye tracking can provide valuable data in distinguishing PD patients from healthy individuals, emphasizing the role of dynamic system features in diagnosis.

Continuing this trend, additional studies have used similar methodologies with high accuracy. Prashanth et al. (2016) used SVM on a dataset from the Parkinson Progression Markers Initiative. This method applies data mining techniques to attributes considered to be early symptoms of PD, such as cognitive disorders, rapid eye movements, sleep behavior disorders, and cerebrospinal fluid measurements [39]. Researchers achieved 0.96 accuracy, 0.97 sensitivity, and 0.95 specificity in distinguishing early PD patients from a control group. This high level of accuracy underlines the effectiveness of data mining techniques in early PD detection, providing a strong case for the use of eye tracking in clinical assessments.

Beyond PD, eye tracking has shown promise in other neurodegenerative conditions as well. In another research study by Vodrahalli et al. (2022), infrared oculography was used with visuo-motor tasks involving rapid reading of 40 single-digit numbers [40]. They used convolutional neural networks (CNNs) as a classifier with widow-based analysis of recording which includes fixations and saccades. The results were discriminated among diseases impacting EM (like PD), diseases associated with vision loss, and healthy controls (81% accuracy compared with the baseline of 33%). This approach showcases further the versatility of eye-tracking technology in diagnosing a range of conditions, highlighting its diagnostic potential.

Similarly, recent research has focused on combining eye tracking with other diagnostic methods. Belan et al. (2023) investigated the effectiveness of an eye-tracking-assisted visual inference language task in differentiating individuals with mild cognitive impairment (MCI) or Alzheimer’s Disease dementia from cognitively unimpaired older adults [41]. The research involved 95 participants, including 49 with MCI, 18 with mild AD dementia, and 28 controls. The authors used a non-parametric repeated measures ANOVA model for verbal answers and a linear mixed model (LMM) or its generalized version for the analysis of eye tracking variables. The results showed significant differences in verbal answers across all diagnostic groups, and eye-tracking parameters successfully discriminated AD from MCI and controls. Analyzing oculomotor behavior alongside language assessments demonstrated increased sensitivity for detecting subtle deficits in the MCI-AD continuum, making it a valuable diagnostic tool. These findings collectively indicate the growing importance of eye tracking in diagnosing NDs, offering a more nuanced and sensitive approach to identifying early stages of diseases like PD and AD (Table 1).

However, eye-tracking has traditionally been considered an expensive research method due to the high cost of commercial eye tracking systems [42]. Fortunately, as presented in the next section, recent advancements have made it possible to develop low-cost remote eye tracking systems that maintain clinically significant parameters.

## 7. Convoluted Neural Networks Allow Cost Optimization of Eye Tracking

The development of a model for classification using disease biomarkers, such as those obtained from eye tracking, is a crucial step in neurodegenerative disease research. Embedding these models into practical environments, particularly through web-based eye-tracking measurements, facilitates their use in healthcare settings.

Interestingly, while web-based eye-tracking has shown effectiveness, it typically exhibits marginally reduced accuracy and increased data variance compared to laboratory-based devices. This limitation highlights the necessity for model enhancement and methodological refinement to ensure practical applicability and accuracy in real-world settings. Addressing this need, recent research has turned to convolutional neural networks (CNNs) for enhancing eye-tracking methods. Thus, the integration of CNNs in eye tracking technology represents a significant advancement, aiming to improve the precision and reliability of these diagnostic tools, thus making them more suitable for clinical applications in neurodegenerative disease diagnostics.

For example, CNNs have been used to enhance the accuracy of gaze estimation on mobile devices by focusing on facial features. Akinyelu et al. (2022) utilized the face component, gaze features were extracted from the eyes, and the shape and location of the eyes were encoded into the network through a 39-point facial landmark component and a Visual Geometry Group (VGG) convolutional neural network [43]. Different experiments were performed, and the experimental result revealed that 39-point facial landmarks can be used to improve the performance of CNN-based gaze estimation models. The researchers achieved the highest eye tracking accuracy of 0.96 and Mean Square Error (MSE) of 0.01. This approach demonstrates the potential of using facial landmarks to refine CNN-based gaze estimation models, significantly improving performance.

Similarly, another study explored the use of CNNs with webcams for eye tracking. Meng et al. (2017) presented a paper where CNNs were employed in conjunction with webcams for an eye-tracking approach that relies on the detection of key eye features, including the inner and outer corners, the center of the upper and lower eyelids, and the center of the iris [44]. These feature points were then used to construct six types of original time-varying eye movement signals, reducing reliance on the iris center, especially in low-quality videos. The final step involved training a Behaviors-CNN using these signals to recognize diverse eye movement patterns. This strategy helped to mitigate errors stemming from basic eye movement-type detection and artificial eye movement feature construction. The researchers conducted experiments with their application across various activities and, to assess performance, a visual activity dataset was created using a webcam, comprising nearly 0.5 million frames gathered from 38 subjects. They achieved the highest results in the “reading” category, with a precision of 0.87 and a recall of 0.89. By training a Behaviors-CNN with these signals, the study mitigated errors from basic eye movement-type detection and feature construction, showcasing the adaptability of CNNs in different settings.

Continuing the trend of CNN integration, Gunawardena et al. (2022) have also compared various CNN models for mobile eye tracking. They compared four modern lightweight CNN models (LeNet-5, AlexNet, MobileNet, and ShuffleNet) in search of optimal performance in real-time eye tracking based on video oculometry [45]. The researchers used four lightweight CNN models (LeNet-5, AlexNet, MobileNet, and ShuffleNet) to assess the performance of gaze estimation on mobile devices using the Gaze Capture dataset. To analyze the feasibility of various inference modes—on-device, edge-based, and cloud-based—they conducted an empirical measurement study, quantifying inference time, communication time, and resource consumption. The MobileNet-V3 in this study outperformed in terms of model accuracy with the lowest training MSE after 60 epochs, which was 0.5 ± 0.01, and also providing the lowest response inference time, 17.4 milliseconds, of all evaluated network architectures. The findings also revealed that while cloud-based inference yields faster predictions, the communication time introduces significant latency, eliminating real-time eye tracking based on cloud hosting. On the other hand, the researchers point out that on-device inference is limited by energy and memory consumption, leaving edge-based solutions as the best solution with reasonable response time, memory usage, and energy consumption for eye-tracking applications on mobile devices.

Finally, Rakhmatulin and Duchowski (2020) provide an in-depth analysis of contemporary techniques for webcam-based gaze tracking, offering practical implementations of popular methods [46]. The focus is on exploring various deep neural network models for online gaze monitoring. A novel eye-tracking approach is introduced, enhancing the effectiveness of deep learning methods. The system employs a dual-coordinate system, determining the position of the face relative to the computer through infrared LED detection and the gaze position is obtained through a CNN and a method involving three objects (left, right, and center) for accurate gaze tracking. The implementation demonstrates practical applications by enabling computer interaction control based on gaze.

These findings highlight the effectiveness of edge solutions by balancing response time, memory utilization, and power consumption in eye tracking applications on mobile devices, representing a significant step in optimizing eye tracking technologies using CNNs (Table 2). Hence, we note that the development of digital diagnostic applications in real-time environments is crucial, especially in telemedicine. 

Importantly, the nuances introduced by different digital environments can significantly impact diagnostic reliability. Moreover, the approach of not storing registration data enhances privacy and reduces data management requirements, aligning with the expectations of medical experts and patients for efficient and secure medical services. 

Therefore, it is vital to consider these digital environments when developing and implementing digital diagnostic tools. The immediate results are not only a convenience but a necessity in telemedicine, underlining the importance of real-time processing capabilities. 

## 8. Digital Environments Impact Diagnostics Speed and Reliability

Immediate results from automated diagnostic processes are expected by both medical experts and patients. This aspect is obviously important in telemedicine. When using automated diagnostic processes, both medical experts and patients expect immediate results. Additionally, thanks to this approach, there can be no need to store the data of the registration itself. Moreover, there is growing interest in non-invasive predictors of Alzheimer’s and Parkinson’s Disease, as seen in the use of webcam-based eye-tracking data for classification.

Therefore, experiments with web-based applications were conducted to demonstrate the feasibility of client-side solutions. For example, Yang et al. in 2021 developed the “WebGazer”, a web-based eye-tracking application that was integrated into a widely used JavaScript library (jsPsych) for behavioral research [47]. The procedure and code were modified to minimize calibration/validation efforts and enhance temporal resolution. Testing this approach with a decision-making study on Amazon MTurk, the researchers successfully replicated in-lab findings on the connection between gaze and choice. Notably, there was minimal degradation in spatial or temporal resolution, demonstrating the feasibility of online web-based eye-tracking in behavioral research. 

Śledzianowski et al. (2023) experimented with disconnecting the software from the hardware capabilities to compensate for the lack of professional equipment in the patient’s household [48,49]. The methodology was executed in an online system utilizing readily available home-grade equipment, yielding results comparable to those obtained with an infrared 60 Hz eye-tracker but with fewer artifacts. The findings indicated that the disparity in EM latency, a crucial parameter for distinguishing patients with Parkinson’s Disease, was 16 ms when compared to a laboratory-grade 1000 Hz eye-tracker. It is expected that this approach will play a role in advancing analytic tools for NDs (especially for PD) within computational health, consequently expediting the development of new preventive measures for such conditions. 

A similar off-eye-tracker approach is presented by Harisinghani et al. (2023) [50]. The study addresses the growing interest in non-invasive predictors of Alzheimer’s Disease by exploring the use of webcam-based eye-tracking data for classification. Their previous successful attempts utilized high-end eye trackers during picture narration and reading tasks. In contrast, this study employs a deep-learning approach to build classifiers using eye-tracking data collected with a webcam. While the webcam gaze classifier does not match the performance of the high-end eye-tracking classifier, it outperforms the majority-class baseline classifier in terms of the AU-ROC. The findings suggest that predictive signals can be extracted from webcam gaze tracking, offering a promising proof of concept for the potential use of this technology as an affordable alternative to high-end eye trackers in AD detection, despite the need for further exploration.

Moreover, eye tracking in Virtual Reality (VR) technology is being actively explored for remote diagnosis of neurodegenerative diseases [51]. For example, Orlosky et al. (2017) deliberate the need to conduct research in a laboratory environment [52]. This research addresses the challenges in early and accurate diagnosis of neurodegenerative conditions, particularly Parkinson’s Disease, stating that current evaluation methods are time consuming, require travel to specialized centers, and may lead to misdiagnosis. The authors present a cost-effective Virtual Reality interface designed for the evaluation and diagnosis of neurodegenerative diseases. Utilizing a VR display with an integrated infrared camera, they created a 3D virtual environment mimicking tasks used in patient evaluations. The virtual tasks were designed to elicit eye movements associated with neurodegenerative diseases. The study involved nine Parkinson’s Disease patients and seven healthy controls, testing the system’s ability to emulate clinical tasks. Eye tracking algorithms and image enhancement were applied to the recorded eye movements, and evaluation by physicians confirmed three out of four abnormalities. The VR interface demonstrated potential for clinical diagnosis, with physicians rating visualizations as potentially useful.

Beyond VR, there is also a focus on using pupil and oculomotor measures as biomarkers in neurodegeneration. O’Callaghan et al. (2022) focus on using pupil and oculomotor measures as biomarkers to detect changes in the locus coeruleus (LC) [53]. The LC role is synthesizing norepinephrine (noradrenaline) and it is involved in physiological responses to stress and panic. It is also involved in various neural processes including attention, memory, and cognitive functions. The LC’s degeneration or dysfunction is associated with several neurodegenerative diseases, including Alzheimer’s and Parkinson’s. Hence, the study involved Parkinson’s Disease patients who underwent a pharmacological challenge with the noradrenergic reuptake inhibitor atomoxetine. Ultra-high field 7T MRI characterized the locus coeruleus, and patients were tested on and off atomoxetine using oculomotor eye-tracking tasks and a learning task with pupillometry. The results indicate that atomoxetine improves cognitive performance and saccadic reaction times, with larger pupil responses correlated with locus coeruleus integrity. 

Hence, the findings suggest that pupil and eye tracking measures serve as effective biomarkers for this system and are sensitive to pharmacological interventions, offering potential implications for the early detection and monitoring of subcortical changes in Alzheimer’s Disease (Table 3). 

Eye movement measurement offers valuable insights. But it is not the only key to understanding neurodegenerative diseases. Here we can expand our perspective to include facial expressions. This aspect, often affected in NDs, plays a key role in interpreting patients’ emotional states, correlates strongly with eye movements, and allows tracking the disease progression.

## 9. Emotional States Estimations by Facial Expressions Can Indicate Hypomimia

An interesting extension of the EM approach is the analysis of the patient’s emotions. The analysis of emotional states in PD with ML methods is not yet common. However, the current findings emphasize a gap in real-world implementations. Emotions can change during neurodegeneration, creating a clear trace of the disease visible, for example in different facial expressions. Here, scientists often mix bio-signals coming from different sources, for example, they study the relationships between the properties of eye movements in various emotional states expressed by facial expressions. 

Various studies have explored this multifaceted approach, offering insights into the diagnostic potential of these methods. Importantly, mature open-source projects like OpenFace provide state-of-the-art results for selected facial action unit (AU) recognition [54]. OpenFace utilizes the Facial Action Coding System (FACS), which objectively measures and categorizes facial expressions by breaking them down into distinct movements known as Action Units [55]. It provides two machine learning models: one for determining the presence of an AU and another for describing its intensity on a 5-point scale. This system allows for a detailed analysis of facial expressions, such as identifying the combination of AUs that comprise the expression of “happiness” or “sadness”, providing meaningful support for research. The contribution of open-source projects like OpenFace is invaluable in research on neurodegenerative diseases. 

Using this tool, Śledzianowski et al. (2021) studied facial emotions (FEs) through the muscle activity model (FACS) and chaos parameters in EMs [56]. The research revealed that parameters associated with chaos exhibited a strong positive correlation with happiness, while linear and noise components were mostly negatively correlated with this emotion. The model proposed simultaneous emotion recognition based on facial landmarks and EMs, providing a significant modification in emotion estimation models. Many nonlinear models have been tested, with the best results achieved by using the K-Nearest Neighbors algorithm with an accuracy of 0.89 with an ROC-AUC score of 0.88, F1 score of 0.89, Precision of 0.85, Sensitivity/Recall of 0.93, and Specificity of 0.82. This study highlights the nuanced relationship between facial expressions and EMs in emotional states, providing a significant modification in emotion estimation models.

Interestingly, further validation was conducted using the Affectiva-MIT Facial Expression Dataset (AM-FED) which included both human and algorithmic classifications [57]. It confirmed that EM chaos emerged as a biomarker of happiness, and it can play a more crucial role than the intensity of specific muscle activity (AU12). It means that true happiness can be detected even in situations when the lower part of a face is covered, which often happened, e.g., during the COVID pandemic. The model is suggested as an extension for estimating happiness based on facial muscle activity, with potential applications in medical analysis of diseases like PD where facial expressions may be affected by hypomimia. This approach demonstrates the potential of using facial tracking for diagnostic purposes in neurodegenerative diseases.

Others also examined the relationship between facial expressions and PD symptoms. Pegolo et al. (2022), addressing hypomimia in PD, aimed to create a quantitative index, termed the Face Mobility Index (FMI), utilizing a face tracking algorithm based on the Facial Action Coding System [58]. The researchers used OpenFace to detect facial landmarks’ positions and extract features. The software considers distances between pairs of facial features to distinguish between healthy individuals and those with PD. The index was also evaluated for emotion classification. The results indicated that FMI effectively quantifies impairment in PD, showing statistically significant differences for all emotions when distances between features are considered and for happiness and anger when FMI is applied. kNN was found to be the optimal technique in the classification with FMI and the results based on the AUC yielded values ranging between 88.9 and 88.4 and F1 scores were between 70.1 and 73. This research contributes to understanding the quantitative aspects of facial impairment in PD, offering new avenues for clinical evaluation.

Moreover, Almutiry et al. (2016) suggest the potential for the automated measurement of day-to-day variations in PD symptoms based on FEs. The research introduces a method to assess facial expressivity for enhancing PD clinical evaluations [59]. Given controversial evidence about PD-related facial impairment, the research aims to explore discriminative and quantitative measures of PD through facial expression analysis. Video clips of eight subjects (four healthy controls, and four PD patients) were recorded over several weeks, focusing on emotion variation. A statistical shape model tracked facial expressions, measuring the amount of expressivity for each subject. The results indicated that movement measures during happiness, disgust, and anger expressions were the most discriminative, with PD patients exhibiting less movement than controls, indeed confirming hypomimia in PD patients. 

The impact of hypomimia on social interaction and quality of life has also been addressed in clinical studies. A comprehensive review examined the role of computational analysis techniques in measuring emotional facial expressions in PD patients [60]. Clinical studies often use the Movement Disorder Society’s Unified Parkinson’s Disease Rating Scale (MDS-UPDRS), with item 3.2 assessing facial expression. Traditional observer-based scales can be time consuming, prompting the exploration of computational analysis techniques for facial expressions. The paper provides a comprehensive review of these techniques for measuring emotional facial expression in people with PD, emphasizing their clinical applications. Additionally, a pilot experimental work on masked face detection in PD is presented, utilizing a deep learning-based model trained on an NVIDIA GeForce 920M GPU, achieving 85% accuracy on testing images. These findings further emphasize the importance of innovative approaches in detecting and understanding the nuances of facial expressions in PD.

In another study, the authors identified a correlation between impaired facial emotion recognition, detectable through online tools, and neuroanatomical changes that necessitate laboratory examinations [61]. The study is focusing on structural changes in the orbitofrontal cortex (OFC) and the amygdala. Using the Iowa Gambling Task (IGT) and Ekman 60 faces test, 24 early PD patients and 24 controls were assessed. Voxel-based morphometry (VBM) analysis of high-resolution structural magnetic resonance images revealed significant gray matter loss in the right amygdala and bilaterally in the OFC in PD patients. Volumetric analyses did not yield significant differences. Correlations between OFC gray matter volume and test performance suggest that OFC and amygdala degeneration are associated with neuropsychological deficits in early-stage PD. This correlation highlights the importance of structural brain changes in understanding the neuropsychological deficits in early-stage PD.

Notably, the recognition of emotions in speech and graphic representation has also been explored. Lu et al. (2022) investigated the recognition of emotions in speech and the graphic representation of expressions gathered during the learning process [62]. They employed an unsupervised Adversarial Autoencoder for feature extraction and utilized convolution neural networks with Bi-directional Long Short-Term Memory (CNN-BiLSTM), achieving an accuracy of 0.99 in emotion classifications. This approach underlines the potential of advanced machine learning techniques in classifying emotions, offering insights into neurodegenerative disease progression.

Moreover, research on EEG features in cross-subject emotion recognition highlights the significance of various EEG parameters in understanding emotions. Li et al. (2018) examined the significance of 18 types of linear and non-linear EEG features in cross-subject emotion recognition, encompassing various channels, brain regions, rhythms, and feature types [63]. Employing SVM with automatic feature selection methods, they verified the potential of exploring robust EEG features in cross-subject emotion. For the dataset containing physiological signals, they attained a mean recognition accuracy of 0.83 (AUC = 0.9). This research demonstrates the potential of EEG features in providing robust biomarkers for emotional states in neurodegenerative diseases.

An interesting direction of research is also the analysis of emotions based on text (sentiment), which in the case of neurodegenerative diseases rather concerns the early phase of the disease or tests before the medical diagnosis of the disease. Such an example is the study presented in “Deep learning approach to text analysis for human emotion detection from big data”, where the authors introduced a Deep Learning-Assisted Semantic Text Analysis (DLSTA) for identifying human emotions in text documents using big data [64]. This method involved an RNN/CNN on text content to generate word embeddings, extract feature vectors, and perform final classification with SVM. For different emotions, they achieved a mean accuracy for prediction of 0.83 and detection between 0.92, with a mean recall of 0.85 and an F-Measure of 0.92. This method’s success in detecting emotions from text highlights the potential for early diagnosis and monitoring of neurodegenerative diseases through linguistic analysis.

The research leveraging machine learning and facial expression analysis, including tools like OpenFace and datasets such as AM-FED, demonstrates significant potential in diagnosing and monitoring neurodegenerative diseases by identifying emotional states and facial mobility impairments (Table 4). This leads us to another important area: digital tools that help to spot signs of early changes in the brain.

## 10. Digital Tools Allow Cognitive Decline Detection

New evidence shows that AI and digital tools are good ways to find signs of brain diseases early. They might help slow down the diseases [65,66]. These tools can spot diseases that cause dementia long before people start showing clear signs of memory or thinking problems. They can help guide changes in how people live and decide who should join medical studies [65,67]. Furthermore, these tools might even help find these diseases before any symptoms show up, and this could lead to new ways to treat the diseases [68]. 

In mild cognitive impairment, AI and virtual reality can be used to create predictive models based on digital biomarkers, enabling early detection and interventions [69,70]. Moreover, AI shows the potential to assist with the detection of early-stage dementia, offering cost-effective and objective methods [71]. Furthermore, an increasing array of Medtech tools are being developed to monitor daily routines and behaviors, capturing early cognitive changes indicative of NDs [72]. 

However, it became evident that the field introduced multiple viewpoints of digital biomarkers that might appear unrelated to or inconsistent with each other, obscuring the clear picture of research perspectives. This critique follows Motahari-Nezhad (2022), who emphasizes the need for high-quality studies and the consideration of methodological criteria and evidence quality [73]. 

As well, Sobolev (2021) highlights the potential of digital biomarkers in predicting and influencing health conditions but also underscores the need for technological integration and the challenges in this area [74]. Similarly, Babrak (2019) discusses the basic differences and similarities between traditional and digital biomarkers and points out the necessity for synchronization and unique feature definition claiming, therefore, that the unclear definition of digital biomarkers, population groups, and their intersection with traditional biomarkers hinders their discovery and validation [22]. Finally, and recently, Alonso et al. (2023) even concluded that there is no consensus about what this emerging term *means* [75]. 

Additionally, it’s interesting to note that the approach to diagnosing Alzheimer’s Disease has evolved over the years. Previously, AD was diagnosed when the patient exhibited both cognitive changes and symptoms related to dementia. However, the US National Institute on Aging and the Alzheimer’s Association (NIA-AA) now defines AD as a diagnosis based solely on biological biomarkers, even if clinical symptoms are not present yet. This means that AD can be diagnosed much earlier than before. The FDA has developed a staging system for AD, which ranges from stage 1 (normal cognition and biomarker evidence of AD) to stages 4–6 (mild, moderate, and severe dementia).

Importantly, these findings collectively underscore the necessity for a coherent definition of those approaches, at least, to reveal the potential of digital biomarkers and AI methods in Medtech applications.

Hence, to characterize the tandem of digital biomarkers and AI tools, the literature suggests using the term “digital phenotyping”, where the assumption is that an individual’s health experience is reflected in the digital traces that they leave behind [76]. These traces are further translated as data collected from everyday interactions with technology like smartphones and wearable devices. This approach is notably transforming the way we understand and detect NDs, leveraging everyday technology to gather valuable health data [77]. 

However, the effectiveness of these digital tools as reliable screening mechanisms remains underdeveloped despite their potential and advancements in the AI field. Those challenges were presented in several studies and projects that explored the use of digital technology in detecting and monitoring neurodegenerative diseases, though with varying degrees of success [78]. Here, we must emphasize the importance of good user experience (UX) in cognitive screening tools, not only for engagement but also for the accuracy and reliability of the collected data.

## 11. User Experience Impacts Data Reliability

The necessity of a good user experience in cognitive screening tools is crucial, not only for user engagement but also for the accuracy and reliability of the data collected. One notable project in this domain is the Altoida initiative, which focuses on identifying digital biomarkers for mild cognitive impairment [79]. 

The Altoida model, based on app data, demonstrated high accuracy (AUC = 0.92) in predicting the transition from MCI to dementia [80]. This accuracy was further validated in subjects with MCI due to AD, corroborated by positive beta-amyloid and imaging biomarkers [81]. This high level of accuracy is significant as it showcases the potential of mobile-based applications in the pre-symptomatic detection of NDs. Altoida’s recognition by regulatory bodies like the FDA and CE further highlights its clinical relevance [82].

Similarly, another study focused on the Smart Aging Serious Game (SASG) demonstrated the effectiveness of digital platforms in cognitive health assessment [83,84]. The project included a virtual reality platform designed for the ecological assessment of mild neurocognitive disorders. With a focus on mild cognitive impairment and vascular cognitive impairment (VCI), conditions associated with a heightened risk of developing dementia, the research aimed to validate SASG’s diagnostic capabilities [84]. 

Importantly, the SASG was successful in identifying the distinct cognitive profiles associated with MCI and VCI, aligning with traditional neuropsychological assessments. An ROC analysis indicated that SASG and MoCA both demonstrated high diagnostic sensitivity and specificity (AUC values greater than 0.80) for identifying VCI versus HC and MCI versus HC. The classification accuracy for distinguishing these groups was high, with RF and LR analyses showing between 75% and 91% accuracy [84]. Hence, the SASG proved to be an effective digital phenotyping tool that aligns with traditional neuropsychological evaluations, capable of early and potentially self-administered assessment of cognitive impairments. 

What is noteworthy in that context is a study by Illiadou et al. (2021) which was conducted with the administration of a self-administered test in a VR (the “Virtual Supermarket Test (VST)”) environment combined with a cheap and commercially available wearable EEG. It is possible to register elevated EEG rhythms in the MCI group when solving tasks in virtual reality, which therefore may be associated with an overall cognitive decline [85]. 

Interestingly, the SASG example further supports the necessity of a good user experience in cognitive screening tools. Here, usability is crucial, not only for user engagement but also for the accuracy and reliability of the data collected. A recent study (2022) by Zygouris et al. evaluated the usability of the Virtual Supermarket Test [86]. Twenty-four older adults with subjective cognitive decline and thirty-three patients with MCI completed the VST and subsequently assessed its usability using the System Usability Scale (SUS). The results were notable, with an average SUS score of 83.11 out of 100 points (SD = 14.6), indicating a high level of user-friendliness. This score is particularly significant as it remained consistent regardless of the participant’s age, educational background, familiarity with touch devices, or MCI diagnosis. Furthermore, there was a notable correlation between the SUS score and VST performance (r = −0.496, *p* = 0.000), suggesting that better usability is associated with more accurate cognitive assessment [86]. 

Therefore, the development of cognitive screening tools like the VST must prioritize UX design [87,88]. Good UX not only facilitates broader accessibility and usability among diverse user groups but also ensures the integrity and validity of the data gathered, which is essential for accurate diagnosis and effective monitoring of conditions like MCI.

Hence, but not surprisingly, user-centered design and evaluation are crucial in clarifying and adjusting the level of detail in web-based decision aids for individuals with MCI [89]. This is important because usability studies have shown that users with MCI require more time and help to complete tasks and that the speed of audio help can significantly impact performance [90]. 

However, these findings have further implications for tools that can detect (with sub-millisecond precision) slowness of reaction time, serving as potential biomarkers for bradykinesia (slowness of movement). Thus, while it is important to make web-based tools easy for people with MCI to use, these tools can also detect slow reaction times (Table 5). This slowness is a key sign of bradykinesia, which is often seen in neurodegenerative diseases.

## 12. Digital Tools Can Detect Slowness of Reaction Time

Interestingly, slower task completion by users with mild cognitive impairment is not just a challenge but somehow obviously an important feature that can be leveraged for early detection.

These findings align with the study by Donoghue et al. (2012), which showed an association between functional mobility, as measured by the Timed Up-and-Go (TUG) test, and various cognitive functions. Their analysis revealed that slower TUG performance is independently associated with poorer global cognition, executive function, memory, and slower processing speed [91]. 

Importantly, simple reaction time (SRT) decline is visible not only in advanced stages of AD or PD but also in individuals with MCI (a risk factor for PD and AD), therefore being possible early digital biomarkers of those diseases. This is supported by a meta-analysis by Andriuta (2019), who analyzed seven selected studies with a total of 327 participants with MCI and 468 healthy controls (HCs); the mean SRT was significantly (*p* = 0.0217) longer in the MCI group (by 11%) than in the HC group [92]. This observation is crucial because slower response times, often consistent with cognitive impairment, can be precisely captured on web platforms.

Web platforms can measure reaction times and task completion speeds in milliseconds, turning into a part of digital phenotype. This finding is further supported by our recent study (2023) that involved collecting cognitive and behavioral data from PD patients and healthy controls [93]. Here, an online platform helped to collect the time between the screen appearing and the participant’s first option selection (instrumental reaction time—IRT) and the time it takes for the participant to click the submit button (time to submit—TTS). The key finding was that IRT and TTS were significantly slower (*p* < 0.001) in the PD group compared to healthy controls, especially in tests like the MoCA and Epworth Sleepiness Scale and beyond the healthy age-related decline of the reaction time [93]. 

Moreover, research in the literature highlights the significant potential of online platforms and mobile devices in measuring reaction times and task completion speeds, key indicators of cognitive and behavioral changes. Already in 2011, Cinaz et al. explored wearable devices as tools for measuring reaction times, particularly in the context of everyday cognitive functioning, and concluded that they are feasible to measure changes in reaction times [94]. That is supported by Burke (2017), who demonstrated that devices like the Apple iPad and iTouch can accurately measure reaction times [95]. Bonnechère (2022) extended this work by using cognitive mobile games to assess the evolution of reaction times across different tasks [96]. These studies collectively underscore the versatility and practicality of digital devices in cognitive monitoring.

This finding aligns with the research by Roos J. Jutten et al. (2022), which investigated fluctuations in reaction time (RT) performance as a marker of early amyloid-related neurodegeneration in preclinical Alzheimer’s Disease. Their study employed computerized cognitive testing to measure intraindividual variability in RT (IIV-RT) over some months, using tasks of varying complexity. They found that greater IIV-RT, especially in complex RT tasks, was associated with steeper cognitive decline in individuals with elevated AD biomarkers [97]. Therefore, incorporating these metrics into clinical assessments can provide valuable insights into the early stages of neurodegenerative diseases, enhancing diagnostic strategies and patient care.

However, Schatz (2015) emphasized the need for careful validation and calibration of these digital tools, particularly tablet-based devices, to ensure accurate reaction time assessments [98]. This highlights an essential consideration in the development and application of these technologies. Nevertheless, when introduced properly, even the casual card game Klondike Solitaire may measure time-related values (like time spent on thinking of a move) that can be useful in distinguishing games played by older people with MCI from their healthy peers (AUC > 0.877), as presented by Gielis (2021) [99].

Therefore, the slower performance seen in PD patients is not a drawback but a useful clue about the disease’s progress, helping build a ‘digital phenotype’ that improves early diagnosis. In line with this, digital versions of tests like the MoCA, Epworth, or TMT can add time-related measurements, making digital tools like user-friendly mobile apps valuable for spotting early signs of diseases like PD or AD (Table 6). Moving to a broader context, web and mobile technologies stand out as affordable options for checking cognitive health.

## 13. Web and Mobile Technology Are Affordable Tools for Screening Cognitive Deficits

Recent findings suggest that in the context of the early detection of cognitive decline, the potential of mobile applications is substantial. As Thabtah (2020) noted, some of these apps employ advanced techniques like machine learning and AI to enhance diagnostic accuracy [100]. This approach expands access to early cognitive health assessments, benefiting a broader population segment, including those in underserved areas.

Consequently, digital applications (utilizing smartphones and wearables) hold significant promise as accessible, affordable, and equitable tools for screening cognitive deficits. Chinner (2018) and Naslund (2017) highlight how these tools, by leveraging widely available technology, become practical even in resource-limited settings and offer an affordable alternative to traditional diagnostic methods [101,102]. These methods are particularly effective in supporting clinical care and promoting treatment adherence, especially in low- and middle-income countries.

Moreover, the web-based cognitive testing approach presented in “cCOG: A web-based cognitive test tool for detecting neurodegenerative disorders” explores the effectiveness of a web-based cognitive tool in detecting mild cognitive impairment and dementia for standardized screening in neurodegenerative disorders [103]. The computerized test battery was based on the three classical cognitive tasks: a modification of the wordlist test, a simple reaction task, and the Trail Making Test. It was divided into seven tasks to complete in approximately 20 min to complete with a keyboard and mouse or a touchscreen. Analyzing clinical data from three European cohorts, including 306 cognitively normal, 120 MCI, and 69 dementia subjects, the study compared the global cognitive scores derived from standard neuropsychological tests. The tool demonstrates accuracies (ROC-AUC) ranging from 0.71 to 0.84 for MCI and 0.86 to 0.94 for dementia when administered both at the clinic and in the home environment. Hence, the results indicate this tool as a promising and cost-effective tool for MCI and dementia detection through home-based cognitive assessments.

Web and mobile technologies, through the use of machine learning and AI in apps and web-based tools, offer affordable, accessible screening options for cognitive deficits, showing promise in the early detection of neurodegenerative disorders with demonstrated effectiveness even in resource-limited settings (Table 7). Understandably, the growing use of mental health applications also brings to the fore challenges concerning evidence-based guidelines and transparency.

## 14. Quality of Digital Health Can Be Enhanced through Standardization

The issue regarding guidelines and transparency is highlighted by Torous (2017), who emphasizes the need for transparency and trust in the evaluation and use of these applications, given the concerns about their proliferation and the lack of established guidelines [104]. That statement was further supported by de Francisco Carvalho (2019), who pointed out the necessity of clear ethical guidelines on how information can and should be used [105]. Thus, while digital applications offer immense potential in the early detection and diagnosis of cognitive deficits, ensuring their effective and ethical implementation requires addressing challenges such as evidence-based validation, transparency, and user trust.

These challenges also include the crucial aspects of data privacy and user engagement. Ensuring data privacy is vital for patient trust and legal compliance, while maintaining user engagement is essential for consistent and reliable data collection. Beyond these practical considerations, ethical implications present additional tension. As Ford, Milne, and Curlewis (2023) discuss, deploying digital biomarkers and AI at scale raises concerns regarding accuracy, bias, and equitable access [106]. This is important because to leverage digital tools effectively in clinical settings, those challenges need to be addressed urgently.

These concerns are critical, as they directly impact the validity and fairness of the diagnostic process. Hence, while digital applications offer immense possibilities in the early detection and monitoring of cognitive health, their successful implementation centers on addressing these multifaceted challenges, ensuring they can be effectively integrated into healthcare practice.

In response, recent studies suggest that a consensus on standards in digital health research could markedly improve the quality of these studies. The lack of consensus on digital outcome measures and their integration into clinical practice and research has been noted by other experts, prompting various proposals for standardization. This is underlined by Bejani et al. (2023), who, moreover, offer strategic insights for the inclusion of patient-centered digital measures in research [38].

Furthermore, the main critique by Jha et al. (2023), termed the “Single Digital Biomarker Hypothesis,” revolves around the tendency of current digital measures to simplify the complexity of PD into a single severity score [107]. The authors argue that PD is a multi-dimensional, multi-etiologic syndrome that cannot be adequately represented by a single number. The nuances of individual patient experiences, which can vary widely in symptoms and progression, are likely to be overlooked by such a reductive approach.

Moreover, Espay et al. (2016) discuss the challenges and opportunities in utilizing technology for PD diagnosis and treatment [108]. This is an outcome of the vast amount of data collected and its limited clinical application. Additionally, challenges include the incompatibility of technology platforms, the need for widespread deployment among elderly patients, and the complexity of translating big data into clinically relevant insights. As a solution, Espay et al. (2019) formed The International Parkinson and Movement Disorders Society Task Force on Technology, which aims to address these issues by promoting the development of open-source and open-hardware platforms for multichannel data capture [109]. The goal is to create adaptable systems for individualized treatment delivery, encouraging early detection, tailored therapy, and subgroup targeting for testing disease-modifying treatments and identifying objective biomarkers for improved longitudinal tracking of PD.

Still, Assunção (2022) argues for the adoption of a more comprehensive framework. This framework would assess and communicate the benefits of early interventions, crucially bridging the gap between the development of disease-modifying therapies (DMTs) and their practical, clinical applications [30].

An additional critical observation within the literature echoed in practical applications is the disappearing patient interest in digital solutions over time. It is hypothesized that such tools may overly focus on the disease rather than the patient, leading to a perceived lack of value and progressing disengagement. As articulated by Bloem et al. (2020), the patient centricity of digital tools is imperative for sustained patient engagement [110].

Hence, while standardized, patient-centric approaches in digital health tools enhance interventions and improve outcomes in neurodegenerative diseases, this might be a good moment to take a step back and reconsider the algorithms we use. To further refine these tools, we should delve deeper into the brain’s underlying mechanisms, drawing inspiration from how our minds work, which, as Turing’s theories and recent psychophysical experiments suggest, might be more about simple rules and pattern recognition than complex, computational-heavy, AI-driven ‘black box’ methods.

## 15. Machine Learning Models Support Diagnosis and Monitoring of NDs

It is interesting to note that the brain’s functioning is often compared to that of a digital computer or a Universal Turing Machine, which processes symbols [111]. However, psychophysical experiments and our ability to recognize complex objects, such as faces, in various contexts and lighting conditions suggest otherwise. This argues against symbolic representation and instead supports the idea that concept representation based on similarities may be a more appropriate model for how the brain works.

Hence, we propose to direct our eyes to Turning’s lesser-known contribution to the field of developmental biology. Turing proposed that natural patterns like stripes, spots, and spirals can arise naturally from the interaction of two or more chemical substances, which he called “morphogens” (that is, the movement of “chemicals” between cells that causes cells to transform/morph into the next “state”) [112]. This research explores how complicated patterns, like those seen in zebra stripes, can emerge from relatively elementary biochemical processes.

Applying this concept to brain development, Turing’s theory suggests that complex structures and patterns in the brain could emerge from simple, preprogrammed rules at the cellular level. This perspective contrasts with the view of the brain as a Universal Turing Machine, which implies a more fixed, predetermined computational process. Importantly, the shift from viewing the brain as a rigid, symbol-processing Universal Turing Machine to a more fluid, self-organizing system, as suggested by Turing’s morphogenetic principles, allows for a more nuanced understanding of cognitive processes.

Interestingly, this approach resonates with the research of Levin et al. (2021) who created the first *living robots*, known as xenobots [113]. Levin’s research explores how cells can self-organize into complex structures and forms using basic rules. Using xenobots, he presents how individual cells self-organize into complex tissues and morphologies. This is important in the context of NDs, because changes in cellular patterns and processes could be detectable before clinical symptoms arise, enabling earlier intervention and potentially more effective treatment. Additionally, understanding how cells communicate and organize themselves to regenerate tissue can inform strategies to promote neural regeneration in neurodegenerative diseases.

Therefore, we suggest that the idea of using logical rules for object classification resonates with Turing’s reaction-diffusion theory and Levin’s work on morphogenesis. Both emphasize the emergence of complex patterns and structures from simpler rules and interactions. In the brain, these ‘simpler rules’ could be the logical rules used in Rough-Set Theory for visual processing.

## 16. Rough Rules Can Implement Visual System Principia

Interestingly, the anatomical and neurophysiological basis of object shape classification and the computational properties of the brain can be described by Rough-Set Theory (RST). Introduced by Pawlak (1982), the RST offers a framework for understanding how the brain processes complex visual information, despite the imprecise nature of concepts representing objects’ physical properties [114,115]. This suggests that concept representation based on similarities rather than symbols may be a more accurate model for how the brain works [116].

The application of the RST in visual classification involves specific neural interactions. The visual classification model is based on the receptive field properties of neurons in different visual areas and uses both feedforward and feedback interactions between them.

- The feedforward pathways use “driver logical rules” to combine properties extracted in each area into hypotheses related to possible objects.
- The feedback pathways use “modulator logical rules” to help change weak concepts of objects’ physical properties into crisp classifications in psychophysical space [117].

**Figure 1:**
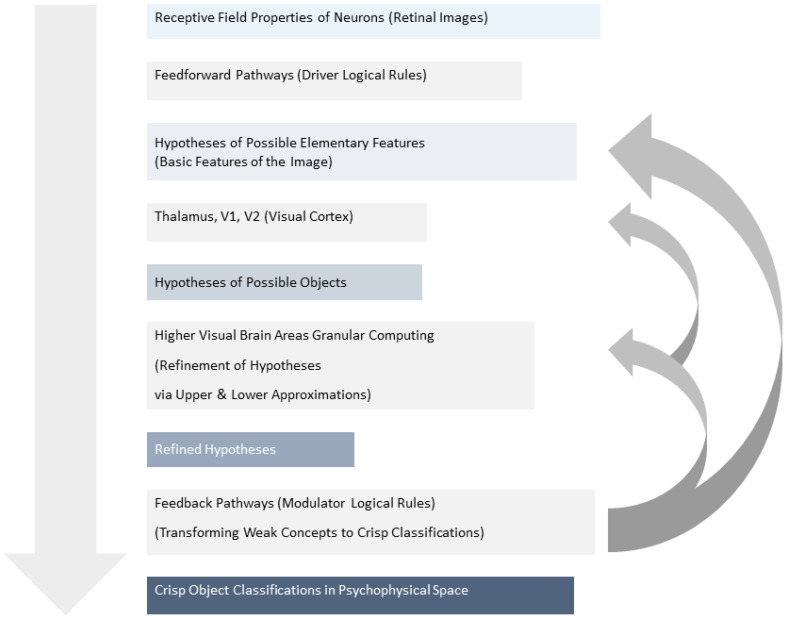
The receptive field properties of neurons fed into both feedforward and feedback pathways. Feedforward pathways use “driver logical rules” to create hypotheses of possible objects, while feedback pathways employ “modulator logical rules” to refine these hypotheses into crisp classifications. Higher visual brain areas then utilize Granular Computing (GrC) to compare upper and lower approximations of retinal images with stored object models, leading to object recognition as the gap between these approximations narrows.

Hence, this process approximates how the brain utilizes logical rules to transform blurred object concepts into clear categorizations, mirroring the principles of the RST (Figure 1).

In higher visual brain areas, the described processes utilize Granular Computing (GrC) to identify upper and lower approximations of the retinal image. These approximations are then compared with different objects’ models (images) stored in the visual cortex. As object recognition or classification progresses, the lower visual areas are tuned to extract the properties of the selected model (modulator logical rules), and the gap between the upper and lower approximations narrows. If the border set becomes empty, the object is successfully recognized. Hence, this approach can be applied to propose various models that approximate the actual (future) cognitive state of tested subjects.

Importantly, this theory is further supported by research on macaques. Przybyszewski et al. (2000) investigated the back-projection pathways from the striate cortex (V1) to the lateral geniculate nucleus (LGN) [118]. These are connections that go from one part of the brain back to an area that supplies input to it. Research shows that these back connections make neuron responses stronger, depending on the contrast of what is seen. These findings revealed that the way neurons in the LGN react to visual stimuli is greatly increased by these back-projection pathways. This happens especially when the contrast in the visuals is high. Hence, it shows that the connections from the striate cortex to the LGN play a big part in how well neurons respond to different levels of contrast and colors in what we see.

Therefore, these new insights fit well with the Rough-Set Theory-based models of visual processing. These predictive models, developed based on clinical studies, reveal various patterns of neurodegenerative diseases, analogous to the visual representation of complex objects in higher visual cortical areas.

However, given the variability in symptoms among patients, it is necessary to have adaptive mechanisms that can accommodate the approximate and flexible nature of these variations. Hence, a critical question remains: how can these mechanisms identify objects or their elements in new conditions, especially in the context of identifying diseases with unseen preliminary indications?

To address this challenge, a novel approach has been proposed, which extends the classical definition of the receptive field (RF) to a fuzzy detector. The properties of the RF are further defined by the computational attributes of the bottom-up and top-down pathways, which compare the stimulus against multiple predictions.

## 17. Fuzzy Detectors as Possible Predictive Models of NDs

By using this new approach, it is possible to detect and recognize objects in different conditions, including those that are unseen. This approach can also be used to identify the presence of neurodegenerative diseases in patients, even when the symptoms vary between individuals. Hence, a fuzzy detector is a promising tool for developing more accurate and effective predictive models for neurodegenerative diseases, which can help to improve diagnosis and treatment.

That is an insightful observation because the utilization of AI methods to generalize and intellectualize patients’ symptoms could be a game-changer in detecting preclinical indications of neurodegenerative diseases such as PD and AD. By creating models based on granular computing approaches, AI can use these models as references to classify potential preclinical indications of ND. This approach could open new possibilities for preventing or curing neurodegenerative brain pathologies, potentially leading to more effective treatments for these devastating conditions.

The aim is to utilize the insights of movement disorder specialists to identify significant attributes of NDs for AI-based classification. Therefore, a project by Przybyszewski et al. (2021) employing a combination of Theory of Mind (ToM) and supervised learning, based on granular computing (GrC) and Rough-Set Theory, seeks to emulate the expertise of movement disorder specialists. This system is based on a computing approach that uses Rough-Set Theory and abstract granules to represent the ToM of many movement disorder experts. It identifies similarities between granules obtained from one group of more advanced PD patients to estimate the disease progression of other patients. While it was found that the accuracy of prediction increased with disease progression, it is noted that divergent sets of granules characteristic of different parts of the brain might degenerate in different ways with disease progression [117]. The exploration of various AI methods further underscores this field’s potential.

To challenge this method, researchers have compared different AI methods, including GrC as implemented in Rough-Set Theory (RST) and fuzzy Rough-Set Theory (FRST) [119]. These methods were compared with other classical machine learning techniques for predicting PD progression, including Nearest Neighbors, Decision Tree, Random Forest, Support Vector Classification, and Gradient Boosting. Dutta and Skowron (2021) have used complex granules (c-granules) to model longitudinal disease development, finding that the RST provides the best estimations of disease progression [120]. The study found that the RST gave the best estimations of disease progressions measured as accuracies, while the FRST gave smaller values of accuracies but better global coverage. Other AI methods gave similar results but only when looking for the disease progression without or with medication separately, indicating their limitations in looking for longitudinal PD progressions [121].

It is fascinating to see the potential of machine learning models, including Rough Sets, in predicting the optimal treatment for NDs. A further example is a study by Przybyszewski et al. (2020) analyzing patients under different treatments, which achieved varying degrees of accuracy, suggesting the potential to discover universal rules for PD progression [122]. The research presents an overall accuracy of 70% for medication visits, 56% for DBS (deep brain stimulation), and 67% and 79% for post-op second and third visits, respectively. This exploration highlights the exciting possibilities of AI and machine learning in advancing our understanding and management of neurodegenerative diseases, potentially leading to more tailored and effective treatments (Table 8).

## 18. Intelligent Granular Computing (IGrC) Can Predict Cognitive Patterns

Cognitive symptoms are more dominant in Alzheimer’s Disease, whereas motor symptoms are more pronounced in Parkinson’s Disease. A study using an IGrC approach examined the relationship between cognitive and motor symptoms in PD, revealing that cognitive changes are independent of motor symptom development [123].

The study involved 47 Parkinson’s Disease patients who underwent eye movement, neurological, and neuropsychological tests in two sessions: S#1—without medications (MedOFF) and S#2 after taking medications (MedON). The patients were divided into two groups: Gr1 (less advanced) and Gr2 (more advanced). By applying different sets of rules to different visits, the researchers found that cognitive changes are independent of the motor symptoms’ development.

It is interesting to note that there seems to be a link between depressive symptoms and neurodegenerative diseases. In the case of Parkinson’s Disease, for example, there is a reduction in the level of the reward hormone dopamine, which can lead to a decrease in positive emotional experiences and an increase in depression. On the other hand, in older individuals (over 65 years of age) who experience depression, there is a higher risk of developing late-onset Alzheimer’s Disease (LOAD). The research aims to evaluate this relationship’s strength.

AI and machine learning, including Rough-Set Theory, have been employed to classify patients’ symptoms in PD. The study involved testing 24 patients with Parkinson’s Disease (PD) who were only receiving medical treatment (BMT-group) and 23 patients with PD who were receiving both medical treatment and deep brain stimulation (DBS-group), as well as 15 older patients who had been receiving DBS treatment for one and a half years (POP-group). The patients were tested every six months (W1, W2, W3) using a range of neurological (disease duration, Unified Parkinson’s Disease Rating Scale), neuropsychological (depression-Beck test, PDQ39, Epworth), and eye movement (reflexive saccadic) tests. Using RST, the researchers were able to identify rules from the BMTW1 data (patients receiving only medical treatment during their first visit) that allowed them to predict UPDRS scores for BMTW2 and BMTW3 with accuracies of 0.765 (0.7 without Beck test of depression result) and 0.8 (0.7 without Beck result), respectively [123].

They were also able to use these rules to predict disease progression (UPDRS) in a group of patients in the DBSW1 group with an accuracy of 0.765. Using the rules generated from the DBSW2 data, the researchers were able to predict UPDRS scores for the DBSW3 (acc. = 0.625), POPW1 (acc. = 0.77), POPW2 (acc. = 0.5), and POPW3 (acc. = 0.33) groups [124]. By adding the depression attribute and using GrC, the researchers were able to make more accurate predictions about disease progression in a range of patient groups compared to predictions made without this attribute (Table 9).

## 19. AI and Machine Learning Can Predict Symptoms and Progression of NDs

Recent findings suggest that AI methods predict cognitive patterns in normal subjects, indicating pre-dementia stages. For example, Przybyszewski et al. (2022) used granular computing rules to classify cognitive data from the BIOCARD study, which has been ongoing for over 20 years with 354 normal subjects. The study’s findings suggest that AI methods can predict patterns in cognitive attributes of normal subjects that might indicate their pre-dementia stage, something that may not be visible to neuropsychologists [125].

Another study based on Biocard data provides a significant advancement in the detection and prediction of Alzheimer’s Disease, utilizing AI methods to identify early cognitive changes. Over 20 years, subjects were evaluated annually to determine their cognitive status—normal, mild cognitive impairment, or dementia. The study used the Clinical Dementia Rating Sum of Boxes (CDRSUM) as a quantitative index for assessing mild dementia and developed rough set rules (RSR) for classification. Researchers classified patients of AD, MCI, and normal, based on their CDRSUM scores. They discovered that some subjects showed signs of potential cognitive impairment or mild dementia that were not evident to neuropsychologists. These findings highlight the capacity of AI methods to detect subtle cognitive changes that might indicate a pre-dementia stage [126].

This approach is a critical step forward in the early detection of AD. By identifying patterns in cognitive attributes among normal subjects, AI methods can reveal early signs of dementia, offering a window for intervention before the condition becomes clinically apparent.

Another BIOCARD study utilizes multi-granular computing to refine the process of classifying cognitive data related to Alzheimer’s Disease, aiming for early detection [127]. Researchers modified the number of attributes used in the BIOCARD study, increasing the variety of granules from five to seven attributes, compared to the constant fourteen attributes used previously. This allowed for a more nuanced comparison of classification results. The focus was also on the interpretability of the rules obtained from different granular levels. By creating rules with varying granularity and algorithms, the researchers aimed to identify classifications that are both complete and consistent across different rule sets. The goal is to develop a more accurate and reliable system for early diagnosis, which is critical for effective intervention. Researchers are testing various models to determine the most effective ones for identifying different stages of diseases like Alzheimer’s and Parkinson’s, and they seek classifications that remain consistent irrespective of the algorithms used [127]. Therefore, the overarching aim is to develop a more precise and reliable diagnostic system for early intervention.

Overall, the above studies highlight the potential of digital biomarkers and AI in detecting the early stages of neurodegenerative diseases like Alzheimer’s and Parkinson’s (Table 10). The integration of digital tools into clinical practice could revolutionize the way we diagnose and treat these conditions, ultimately improving the quality of life for millions of people worldwide.

## 20. Conclusions

AI and digital tools promise to facilitate the early detection of neurodegenerative diseases, potentially leading to earlier interventions that could slow disease progression. Studies are being conducted to validate the efficacy of digital biomarkers and AI-based predictive models in identifying early-stage neurodegenerative diseases. These technologies could lead to more personalized treatment plans based on individual data patterns.

This review covers the use of digital phenotyping technologies to capture behavioral information relevant to neurological diseases. Digital endpoints could enhance the precision of clinical trials, aiding in patient stratification and the detection of treatment effects. However, the review also points out the need for a standardized approach to study design to allow for meaningful interpretation of the data collected from various studies.

This standardization would help in comparing results across studies and improving the understanding of neurodegenerative disease trajectories. Interestingly, despite reported advancements in healthcare solutions, it was found that the current digital and mobile health (mHealth) applications are in urgent need of improved functionalities to assist in both patient care management and early diagnosis of NDs.

Nevertheless, the findings presented in this paper support the idea that telemedicine solutions could lead to earlier identification of at-risk individuals and may be more sensitive to disease progression, which is beneficial for discovering disease-modifying treatments. Within this framework, sensors emerge as a fundamental component, highlighting the effectiveness of digital phenotyping in enhancing disease characterization and monitoring. The integration of clinical scales, imaging, biosamples, and digital tools is suggested as the most effective approach for characterizing and monitoring disease [33].

However, the role of digital tools in improving care, research, and outcomes for patients with movement disorders suggests that their full potential is yet to be realized. The care model is perceived as being reactive rather than proactive, with an inadequate response to complex issues due to a lack of disease-specific expertise and underutilization of non-pharmacological treatments.

Moreover, treatment plans are usually more disease centric rather than patient centric, not fully considering the needs and preferences of the individuals affected by these conditions. Consequently, patients may find themselves excluded from the clinical decision-making process, leaving a gap that must be addressed urgently to bring truly meaningful research outcomes.

## Figures and Tables

**Table 1:** List of sensors and their respective domains and metrics.

Sensor	Metrics	Sense—Domain	Results	Reference
Video-based eye tracking	Saccades, pupil, blink behavior	PD detection	Sensitivity: 0.83, Specificity: 0.78, ROC-AUC: 0.88	[37]
Video-based eye tracking	Smooth pursuit eye movements, entropy/regularity, self-similarity	PD detection	Accuracy: 0.74, Sensitivity: 0.73, Specificity: 0.74	[38]
Video-based eye tracking	Cognitive disorders, rapid eye movements, sleep behavior disorders, cerebrospinal fluid measurements	Early PD detection	Accuracy: 0.96, Sensitivity: 0.97, Specificity: 0.95	[39]
Infrared oculography	Fixations, saccades	Distinguishing neurodegenerative diseases	Accuracy: 81% (compared to 33% baseline)	[40]
Eye tracking with visual inference language task	Oculomotor behavior, verbal answer analysis	Differentiating MCI/AD dementia from controls	Significant discrimination between AD, MCI, and controls	[41]

**Table 2:** List of sensors and their respective domains and metrics.

Sensor	Metrics	Sense—Domain	Results	Reference
Facial features and gaze estimation	Facial landmarks, gaze features	Eye tracking accuracy enhancement	Highest accuracy: 0.96, MSE: 0.01	[43]
Webcam-based eye tracking	Key eye features (corners, eyelids, iris center), eye movement signals	Eye movement pattern recognition	Precision: 0.87, Recall: 0.89	[44]
Mobile eye tracking	Lightweight CNN models (LeNet-5, AlexNet, MobileNet, ShuffleNet)	Gaze estimation on mobile devices	Best model accuracy (MobileNet-V3): Training MSE: 0.5 ± 0.01, Response time: 17.4 ms	[45]
Webcam-based gaze tracking	Dual-coordinate system, deep neural network models	Online gaze monitoring	Enhanced accuracy and practicality for computer interaction control	[46]

**Table 3:** List of sensors and their respective domains and metrics.

Sensor	Metrics	Sense—Domain	Results	Reference
Web-based eye tracking (WebGazer)	Spatial/temporal resolution	Behavioral research	Minimal degradation in resolution, replication of in-lab findings	[47]
Online system with home-grade equipment	EM latency	Parkinson’s Disease diagnostics	Comparable results to infrared eye-tracker with less artifacts, latency disparity: 16 ms	[48,49]
Webcam-based eye tracking	AU-ROC	Alzheimer’s Disease classification	Outperforms majority-class baseline classifier, indicates potential for AD detection	[50]
VR with integrated eye tracking	Eye movement analysis	Evaluation of neurodegenerative diseases	Emulates clinical tasks, confirmed abnormalities, potential for clinical diagnosis	[52]
Pupillometry and oculomotor tasks	Locus coeruleus integrity, cognitive performance	Biomarkers for neurodegeneration	Improved cognitive performance and saccadic reaction times with atomoxetine	[53]

**Table 4:** List of sensors and their respective domains and metrics.

Sensor	Metrics	Sense—Domain	Results	Reference
OpenFace	Facial Action Coding System (FACS)	Facial expressions analysis	State-of-the-art results for facial action unit recognition. Provides machine learning models for AU presence and intensity.	[54,55]
Facial Landmarks and EM	Chaos parameters, k-Nearest Neighbors (KNN) algorithm	Emotion estimation models	Strong correlation of chaos parameters with happiness. High accuracy (0.89) and ROC-AUC score (0.88) for emotion recognition.	[56]
AM-FED Dataset	Machine learning, chaos as a biomarker	Happiness estimation	Confirmed EM chaos as a biomarker for happiness, crucial even when the lower face is covered.	[57]
Face Mobility Index (FMI)	Face tracking, kNN	Facial impairment in PD	Statistically significant differences in facial impairment between healthy individuals and PD patients. AUC values between 88.9 and 88.4, F1 scores between 70.1 and 73.	[58]
Video Clips	Statistical shape model	Day-to-day variations in PD symptoms	Highlighted hypomimia in PD patients through decreased movement in expressions of happiness, disgust, and anger.	[59]
Computational Analysis	Movement Disorder Society’s Unified Parkinson’s Disease Rating Scale (MDS-UPDRS)	Emotional facial expressions in PD	Reviewed computational techniques for measuring emotional facial expressions, with a deep learning model achieving 85% accuracy in masked face detection.	[60]
Iowa Gambling Task and Ekman 60 Faces Test	Voxel-based morphometry (VBM)	Neuropsychological deficits in PD	Correlation of OFC and amygdala degeneration with neuropsychological deficits in PD patients.	[61]
Adversarial Autoencoder and CNN-BiLSTM	Emotion recognition in speech and graphic representations	Emotion recognition	Achieved an accuracy of 0.99 in emotion classifications, demonstrating the effectiveness of advanced machine learning techniques.	[62]
EEG Features	SVM with automatic feature selection	Cross-subject emotion recognition	Mean recognition accuracy of 0.83 (AUC = 0.9), highlighting the potential of EEG features in emotional state biomarkers.	[63]
Text Analysis (DLSTA)	Deep Learning-Assisted Semantic Text Analysis	Emotion detection from text	Mean accuracy for emotion prediction of 0.83, with detection accuracy up to 0.92 and mean recall of 0.85.	[64]

**Table 5:** List of sensors and their respective domains and metrics.

Sensor	Metrics	Sense—Domain	Results	Reference
Altoida App	Digital biomarkers	Mild cognitive impairment (MCI) detection	AUC = 0.92	[79,80,81]
VR (Smart Aging Serious Game-SASG)	Cognitive health assessment	MCI and Vascular Cognitive Impairment (VCI) diagnosis	Diagnostic sensitivity and specificity (AUC > 0.80), Accuracy: 75–91%	[83,84]
VR (Virtual Supermarket Test) and Wearable EEG	EEG rhythms	Cognitive decline in MCI	Elevated EEG rhythms associated with cognitive tasks in VR	[85]
VR (Virtual Supermarket Test) and System Usability Scale (SUS)	Usability assessment	Usability and cognitive assessment accuracy	SUS score: 83.11/100, Correlation between SUS score and VST performance: r = −0.496	[86]

**Table 6:** List of sensors and their respective domains and metrics.

Sensor	Metrics	Sense—Domain	Results	Reference
Timed Up-and-Go (TUG) test	Functional mobility, global cognition, executive function, memory, processing speed	Cognitive impairment detection	Slower TUG performance associated with poorer cognitive functions	[91]
Web Platforms	Simple reaction time (SRT)	Early detection of cognitive decline	MCI group showed 11% longer SRT than healthy controls (*p* = 0.0217)	[92]
Online Platforms	Instrumental reaction time (IRT), time to submit (TTS)	Parkinson’s Disease cognitive and behavioral data	IRT and TTS significantly slower in PD group (*p* < 0.001)	[93]
Wearable Devices and Mobile Devices	Reaction times in daily cognitive functioning	Cognitive monitoring	Wearables and devices like iPad/iTouch feasible for measuring reaction times	[94,95]
Cognitive Mobile Games	Evolution of reaction times across tasks	Cognitive health monitoring	Mobile games used to assess and monitor reaction time changes	[96]
Computerized Cognitive Testing	Intraindividual variability in reaction time (IIV-RT)	Early amyloid-related neurodegeneration	Greater IIV-RT associated with steeper cognitive decline in preclinical AD	[97]
Tablet-based Devices	Reaction time assessment	Validation and calibration of digital tools	Need for careful validation to ensure accurate assessments	[98]
Digital Card Game (Klondike Solitaire)	Time spent on thinking of a move	Differentiating cognitive impairment	Can distinguish MCI from healthy peers (AUC > 0.877)	[99]

**Table 7:** List of sensors and their respective domains and metrics.

Sensor	Metrics	Sense—Domain	Results	Reference
Mobile applications	Machine learning and AI techniques	Early detection of cognitive decline	Enhances diagnostic accuracy, expands access	[100]
Smartphones and wearables	Accessibility and affordability	Screening cognitive deficits	Practical in resource-limited settings, supports clinical care	[101,102]
Web-based cognitive testing (cCOG)	Wordlist test, simple reaction task, Trail Making Test	Detecting MCI and dementia	ROC-AUC: 0.71–0.84 for MCI, 0.86–0.94 for dementia	[103]

**Table 8:** List of sensors and their respective domains and metrics.

Sensor	Metrics	Sense—Domain	Results	Reference
Theory of Mind (ToM) and Granular Computing (GrC)	Granules representing expertise	PD progression prediction	Accuracy increases with disease progression; identifies granule differences in brain degeneration	[117]
AI Methods Comparison (GrC, RST, FRST)	Disease progression modeling	PD progression prediction	RST showed best accuracy; FRST provided better global coverage; limitations in longitudinal progression with other AI methods	[120]
Machine Learning Models	Treatment outcome prediction	Optimal treatment for NDs	Accuracies: 70% for medication, 56% for DBS, 67% and 79% for post-op visits	[122]

**Table 9:** List of sensors and their respective domains and metrics.

Sensor	Metrics	Sense—Domain	Results	Reference
IGrC Approach	Cognitive and motor symptoms	PD symptom classification	Cognitive changes independent of motor symptoms development	[123]
AI and Machine Learning (RST)	Neurological, neuropsychological, eye movement tests	PD symptom classification and depression’s impact	UPDRS prediction accuracies: BMTW2 = 0.765, BMTW3 = 0.8, DBSW1 = 0.765, DBSW3 = 0.625, POPW1 = 0.77, POPW2 = 0.5, POPW3 = 0.33 (improved with depression attribute)	[123]

**Table 10:** List of sensors and their respective domains and metrics.

Sensor	Metrics	Sense—Domain	Results	Reference
Granular Computing Rules	Cognitive data classification	Pre-dementia stage detection	AI methods predicted pre-dementia stages in normal subjects from BIOCARD study	[125]
Clinical Dementia Rating Sum of Boxes (CDRSUM) and rough set rules (RSR)	Early cognitive changes	Alzheimer’s Disease detection	Identified subjects with potential cognitive impairment not evident to neuropsychologists	[126]
Multi-Granular Computing	Cognitive data related to Alzheimer’s Disease	Early detection of Alzheimer’s Disease	Developed more accurate and reliable system for early diagnosis with varying granularity	[127]

## Data Availability

Data are contained within the article.
